# Paediatric‐onset autoimmune cytopenia: How can we reduce the long‐term mortality?

**DOI:** 10.1111/bjh.70599

**Published:** 2026-06-09

**Authors:** Nathalie Aladjidi, Thierry Leblanc, Helder Fernandes, Capucine Picard, Jacinta Bustamante, Frédéric Rieux‐Laucat, Mathieu Fusaro, Maud Tusseau, Charlotte Durand‐Teyssier, Emma Cohendy, Jérôme Granel, Stéphane Ducassou, Agathe Escudier, Despina Moshous, Marc Michel, Bertrand Godeau, Sébastien Héritier, Mony Fahd, Brigitte Bader‐Meunier, Alain Fischer, Guy Leverger, All collaborators from the French Paediatric Haemato‐Immunologic CEREVANCE Network

**Affiliations:** ^1^ CEREVANCE, CEREDIH, Pediatric Hemato‐Immunology, CIC1401, INSERM CICP Bordeaux University Hospital Bordeaux France; ^2^ CEREVANCE, Pediatric Onco‐Hematology Robert Debré University Hospital, AP‐HP Paris France; ^3^ Study Center for Primary Immunodeficiencies, CEREDIH, Necker‐Enfants Malades Hospital, AP‐HP University of Paris Cité Paris France; ^4^ Laboratory of Human Genetics of Infectious Diseases, Inserm U1163, Imagine Institute University of Paris Cité Paris France; ^5^ Immunology Department Laboratory, INFINITy, Toulouse Institute for Infectious and Inflammatory Diseases, INSERM, U1291, CNRS U5051 University Toulouse III Toulouse France; ^6^ Department of Medical Genetics, Centre International de Recherche en Infectiologie, Inserm, U1111, CNRS, UMR5308, ENS de Lyon University of Lyon Lyon France; ^7^ Pediatric Immunology, Hematology and Rheumatology, Necker‐Enfants Malades Hospital, APHP Institut Imagine and Université Paris Cité Paris France; ^8^ Department of Internal Medicine, CERECAI Créteil University Hospital Créteil France; ^9^ CEREVANCE, Pediatric Onco‐Hematology, Armand‐Trousseau University Hospital, UMR‐S398 Sorbonne University Paris France; ^10^ Department of Paediatric Hematology‐Immunology and Rheumatology Necker‐Enfants Malades Hospital, AP‐HP, RAISE Paris France; ^11^ Collège de France Paris France

**Keywords:** autoimmune haemolytic anaemia, children, Evans syndrome, immune thrombocytopenia

To the Editor,

Paediatric‐onset autoimmune cytopenia (AIC) is a group of rare and heterogeneous disorders. While mortality in immune thrombocytopenia (ITP) and autoimmune neutropenia (AIN) remains notably low, autoimmune haemolytic anaemia (AIHA) and Evans syndrome (ES) continue to carry high mortality rates of 10%–30%, particularly in adults.^1^, ^2^, ^3^ In 2009, primary ITP was defined as isolated thrombocytopenia without underlying causes or associated immune disorders, with bleeding as the main clinical concern. All other forms of immune‐mediated thrombocytopenia were classified as secondary ITP, but without further specification.^4^ Since then, clinical and therapeutic data on ITP, AIHA, AIN and ES have accumulated across paediatric and adult series. The term ‘secondary AIC’ is widely used, but definitions remain heterogeneous and non‐consensual, conflating diverse conditions as infections, malignancies and autoimmune or autoinflammatory manifestations and strictly causative conditions as transplantation, drug exposure, systemic lupus erythematosus (SLE) or primary immune deficiency/dysregulation (PIDD).^5^, ^6^, ^7^ Following an AIC diagnosis, patients may experience intermittent or continuous flare‐ups and diverse clinical or biological immunopathological manifestations (IMs) throughout life, even after prolonged remission. Robust genetic tools now enable more precise classification of AIC,^8^ which is critical for informing families, guiding personalized care and evaluating established and emerging therapies.^5^ Leveraging the French prospective OBS'CEREVANCE cohort of paediatric‐onset AIC, active for more than two decades, we share our refined definitions to further delineate paediatric‐onset AIC and improve management strategies and long‐term prognosis and quality of life.^1^, ^2^, ^8^, ^9^

Since 2004, French paediatricians from 30 centres prospectively enrol patients diagnosed before the age of 18 with chronic ITP, AIHA and ES into the OBS'CEREVANCE national prospective observational cohort (NCT05937828), following appropriate informed consent. Patients are monitored in routine practice and regularly screened for associated IM and documented infections according to national guidelines; follow‐up data are continuously recorded in the database, including after adulthood. Genetic tools have steadily improved, with Sanger sequencing in the 1980s, next‐generation sequencing (NGS) panels and whole‐exome sequencing (WES) in the 2010s and whole‐genome sequencing (WGS) in the 2020s.^10^ At each follow‐up, AIC are classified into three groups: primary, likely secondary or confirmed secondary. AIC status is coded at every visit, with continuous complete remission (CCR) representing the most favourable outcome (Table S1). Continuous variables were presented as median (range). Overall survival (OS) was defined as the time between initial AIC diagnosis and death, analysed using the Kaplan–Meier method and compared across subgroups with the log‐rank test; *p* < 0.05 was taken to indicate statistical significance. Statistical analyses were performed using RStudio (Boston, MA) and Prism (San Diego, CA).

From 2004 to 2025, 2196 patients with paediatric‐onset AIC diagnosed from 1978 were enrolled by 30 French paediatric centres in the OBS'CEREVANCE cohort. The median follow‐up was 5.4 years (0–42.9). Clinical and biological IM were diagnosed before or after AIC onset in 955 patients (43%), leading to confirmed SLE (*n* = 94, SLICC criteria) or genetic PIDD (*n* = 119, pathogenic validated variants) diagnoses (Table 1). The main IM were non‐malignant lymphoproliferation, positive anti‐nuclear antibodies (ANA) and hypogammaglobulinaemia; 16 patients (0.7%) developed cancer. At the last follow‐up, patients were classified as primary, likely secondary or confirmed secondary in 57%, 32% and 12% of cases respectively. AIC status was CCR in 29%. Of the 1344 living patients (63%) who had reached adulthood (≥18 years), 20% transitioned to follow‐up from paediatric to adult French referral centres.

**TABLE 1:** Context and outcome in 2196 patients enrolled in the national OBS'CEREVANCE cohort for paediatric‐onset autoimmune cytopenia, 2004–2025, as of 19 December 2025.

	Total *n* = 2196	cITP *n* = 1204	AIHA *N* = 573	ES *N* = 419
Demography				
Median age at first AIC, year (min–max)	6.3 (0.0–17.9)	7.6 (0.0–17.6)	3.9 (0.1–17.9)	7.3 (0.1–17.5)
Sex, ratio M/F (*n*)	0.9 (1011/1185)	0.8 (533/671)	1.2 (315/258)	0.6 (163/256)
Familial anamnesis				
Parental consanguinity, % (*n*)	8.3 (87/1050)	7.7 (32/417)	7.2 (26/363)	11.9 (32/270)
IM in first‐degree relatives, % (*n*)	29 (435/1522)	29 (226/780)	24 (101/424)	34 (108/318)
Follow‐up				
Median follow‐up, year (min–max)	5.4 (0–42.9)	5.9 (1–31.6)	3.0 (0–36.1)	9.0 (0.1–42.9)
Total patient‐years of follow‐up	15 878	8868	2735	4275
Patients with <1 year of follow‐up, *n* (%)	144 (7)	0 (0)	125 (22)	19 (4)
Patients lost to follow‐up,^a^ *n* (%)	1086/1800 (60)	630/1093 (58)	270/370 (73)	186/337 (52)
Classification at the last follow‐up				
Primary, *n* (%)	1241/2196 (57)	785/1204 (65)	374/573 (65)	82/419 (20)
Likely secondary, *n* (%)	699/2196 (32)	353/1204 (65)	124/573 (22)	222/419 (54)
Confirmed secondary, *n* (%)	256/2196 (12)	66/1204 (5)	75/573 (13)	115/419 (27)
Post‐transplantation, post‐drugs, *n*	43	5	31	7
SLE,^b^ *n*	94	50	14	30
PIDD,^c^ *n*	119	11	30	78^d^
Treatments				
Patients receiving second‐line therapy, *n* (%)	1092/2196 (50)	597/1204 (50)	194/573 (34)	304/419 (73)
Number of patients with splenectomy, *n* (%)	219/2196 (10)	144/1204 (12)	23/573 (4)	52/419 (12)
Outcome				
Cancer,^e^ *n*	16	2	6	8
Deaths, *n* (%)	64 (0.7)	5 (0.4)	21 (4)	38 (9)
CCR at the last follow‐up, *n* (%)	607/2121 (29)	277/1160 (24)	236/553 (43)	94/408 (23)

Abbreviations: AIC, autoimmune cytopenia; AIHA, autoimmune haemolytic anaemia; CCR, continuous complete remission; cITP, chronic immune thrombocytopenia; ES, Evans syndrome; IM, immunopathological manifestation; PIDD, primary immunodeficiency and/or dysregulation; SLE, systemic lupus erythematosus.

^a^ Patients lost to follow‐up were defined as patients alive, not declared cured by their referring physician, diagnosed before 19 December 2023, and without any informative follow‐up in the database for more than 2 years (last entry prior to 19 December 2023).

^b^ So far, to the best of our knowledge, no case of monogenic SLE.

^c^ Guidelines for genetic studies, see Table S1. In the observational real‐life conditions of this data collection, from more than 100 centres on a period of five decades, we are not able to present the exact number of patients who underwent genetic studies looking for a PIDD when they were not conclusive, nor to detail the tools used.

^d^ 11/419 patients with pES had Autoimmune Lymphoproliferative Syndrome (ALPS), del22q11.2, Kabuki syndrome known before inclusion in the cohort and in a systematic exome trio ongoing study (NCT03912129), in 67/203 (33%) patients, pES revealed a genetically confirmed PIDD.

^e^ Lymphoma (*n* = 6), Hodgkin lymphoma (*n* = 5), sarcoma (*n* = 2), juvenile myelomonocytic leukaemia (*n* = 1), thyroid carcinoma (*n* = 1), melanoma (*n* = 1).

Sixty‐four patients died (2.9%) at a median age of 15.2 years (0.4–41.4), with a median delay of 9 years (0–38) after initial AIC diagnosis; 27 (42%) were older than 18 years. For the entire cohort, the 20‐year OS was 89% (Figure 1). OS was significantly lower in non‐primary than in primary cases (*p* < 0.0001; 20‐year OS 84.6% [95% confidence interval (CI) 78.2%–89.2%] vs. 98.2% [95% CI 94.8%–99.4%]). Excluding the 43 patients with AIC following haematopoietic stem cell transplantation (HSCT) or solid organ transplantation, OS was significantly lower in ES than in isolated AIC (*p* < 0.0001; 20‐year OS 92% [95% CI 88.6%–95.4%] vs. 99% [95% CI 98.2%–100%]).

**FIGURE 1:**
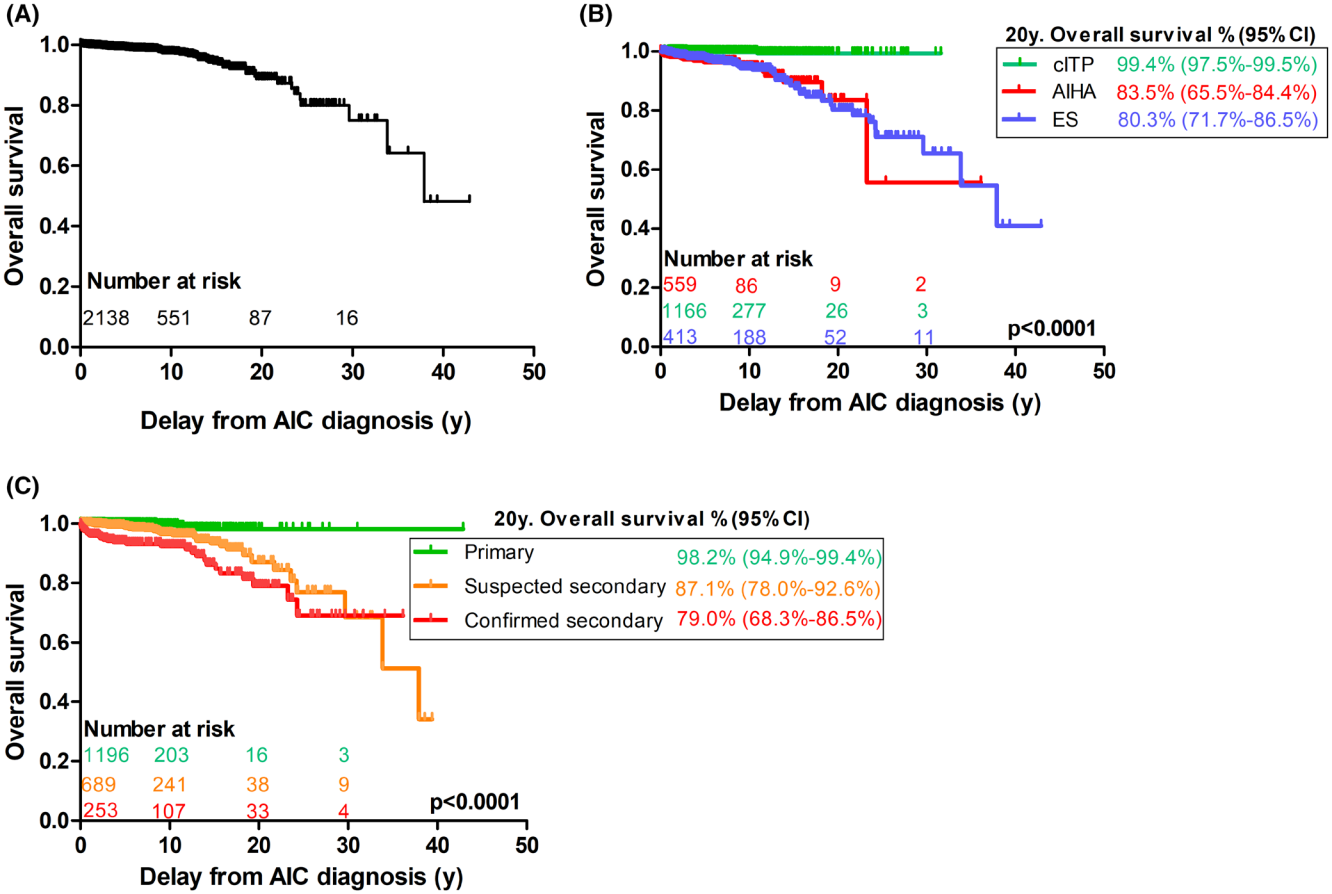
Overall survival in the national OBS'CEREVANCE cohort for paediatric‐onset autoimmune cytopenia: (A) overall population (*n* = 2138, as for 58 patients, the date of initial diagnosis was missing); (B) according to the type of cytopenia cITP, AIHA or ES; (C) according to the classification as primary, suspected secondary and confirmed secondary. AIHA, autoimmune haemolytic anaemia; cITP, chronic immune thrombocytopenia; ES, Evans syndrome.

For rare diseases, prospective observational cohorts provide valuable evidences for clinical practice and carry inherent strengths and limitations. The quality of the OBS'CEREVANCE database relies on the unbiased commitment of each participating clinician. The co‐ordinating team ensures continuous tracking of patient care pathways (relocation, transition) to minimize loss to follow‐up. For example, a second AIC in sequential ES^2^ or complete SLE in children with ANA + AIC^9^ may develop up to 15 years after initial diagnosis, underscoring that studies with shorter follow‐up may fail to capture the full clinical reality. All data are transferred from medical records to a centralized, quality‐controlled platform. To prevent arbitrary or biased coding of IM classifications (ANA, allergy, treatment‐linked lymphopenia or hypogammaglobulinaemia), data are reviewed on a case‐by‐case basis. Considering missing data (e.g. negative or inconclusive historic genetic testing), percentages are calculated using the denominator of available data. Therefore, this national experience is able to provide unique insight into the very long‐term course of paediatric‐onset AIC.

Paediatric‐onset ES continues to carry an unacceptably high mortality rate of 9%, persisting well beyond childhood, and the underlying context is more severe than in isolated chronic immune thrombocytopenia (cITP) or AIHA.^2^ With our classifications and follow‐up, in a pilot study on 80 patients, 80% of ES patients experienced IM, 7% met complete SLE criteria, 40% of explored patients carried pathogenic variants in PIDD genes and 75% required multiple second‐line therapies.^2^, ^8^ For multilineage AIC, aiming to reduce this burden, we recommend genetic testing with appropriate tools within the first 3 months after diagnosis and structured transition programmes with multidisciplinary approaches to prevent loss to follow‐up in adulthood. Given the availability of numerous targeted therapies for immune dysregulation, early identification of the underlying pathophysiological pathway is essential to avoid nonspecific and potentially harmful treatments such as splenectomy.^5^

With systematic evaluation for associated IM and very long‐term follow‐up, our data show that half of patients do not have purely primary AIC limited to thrombocytopenia, anaemia or neutropenia, and that long‐term mortality is significantly higher in non‐primary cases. For 71% of patients with unmet therapeutic needs (no CCR), the major challenge is early recognition of diseases requiring limited ‘wait‐and‐see’ approaches or first‐line strategies, or second‐line strategies such as hydroxychloroquine,^9^ genetic‐based targeted immunomodulation or HSCT.^5^ It is crucial to track IM, as they serve as red flags for suspected PIDD.^11^ Efforts to identify diagnostic clusters or scores are hindered by overlapping phenotypes.^7^ Extensive genetic studies raise medico‐economic concerns. Accurate interpretation of variants requires case‐by‐case multidisciplinary review, integrating clinical data, in silico criteria, RNA and functional assays and familial segregation, particularly in cases of variants of uncertain significance (VUS) or novel pathogenic pathways. It is essential to determine with functional validation whether a variant is causative to support the therapeutic strategy and genetic counselling. This complexity will increase with recognition of oligogenic diseases, somatic variants,^12^ epigenetic mechanisms,^13^ somatic genetic rescues^14^ and protective co‐variants in monogenic diseases.^15^

Although paediatric‐onset AIC are often considered benign, their long‐term burden in adulthood should not be underestimated. For a subgroup, genetic studies have been demonstrated to be helpful. The transition period must be carefully organized by paediatric and adult teams to prevent loss to follow‐up and to improve health outcomes in adulthood.

## AUTHOR CONTRIBUTIONS

**Thierry Leblanc:** Writing – original draft; writing – review and editing; validation; investigation; supervision. **Jacinta Bustamante:** Investigation; writing – review and editing. **Nathalie Aladjidi:** Conceptualization; methodology; investigation; writing – review and editing; writing – original draft; validation; supervision. **Capucine Picard:** Investigation; writing – review and editing. **Helder Fernandes:** Conceptualization; investigation; methodology; formal analysis; data curation; supervision; writing – original draft; writing – review and editing; validation. **Mathieu Fusaro:** Investigation; writing – review and editing. **Emma Cohendy:** Investigation; writing – review and editing. **Maud Tusseau:** Investigation; writing – review and editing. **Charlotte Durand‐Teyssier:** Investigation; writing – review and editing. **Jérôme Granel:** Investigation; writing – review and editing. **Frédéric Rieux‐Laucat:** Investigation; writing – review and editing. **Stéphane Ducassou:** Investigation; writing – review and editing. **Mony Fahd:** Investigation; writing – review and editing; validation; supervision. **Brigitte Bader‐Meunier:** Investigation; writing – review and editing; validation; supervision. **Despina Moshous:** Investigation; writing – review and editing. **Sébastien Héritier:** Investigation; writing – review and editing; validation; supervision. **Alain Fischer:** Investigation; writing – review and editing; validation; supervision. **Agathe Escudier:** Investigation; writing – review and editing. **Marc Michel:** Investigation; writing – review and editing. **Bertrand Godeau:** Investigation; writing – review and editing. **Guy Leverger:** Investigation; writing – review and editing; writing – original draft; validation; supervision.

## FUNDING INFORMATION

This CEREVANCE network has been supported since 2001 by the AFSE, ARMHE and O'CYTO patient associations; since 2007 by the French Ministry of Health (Rare Disease Plans 2007, 2017 and 2023); and since 2014 by the French Filière Maladies Rares Immuno‐Hématologiques (MARIH). We especially thank HF, the data manager, methodologist and statistician for this database for the last 15 years. Genetic immunological studies were supported by the Institut National de la Sante et de la Recherche Medicale (INSERM) and by government grants managed by the Agence National de la Recherche as part of the ‘Investment for the Future’ programme (Institut Hospitalo‐Universitaire Imagine, grant ANR‐10‐IAHU‐01, Recherche Hospitalo‐Universitaire, grant ANR‐18‐RHUS‐0010), the Centre de Reference Deficits Immunitaires Hereditaires (CEREDIH), the Agence National de la Recherche (ANR‐14‐CE14‐0026‐01 ‘Lumugene’; ANR‐18‐CE17‐0001 ‘Action’; ANR‐21‐CE17_00444 ‘Predict JIA’, ANR‐22‐CE15‐0047‐02 « BREAK‐ITP;) the Fondation pour la recherche Medicale (FRM: EQU202103012670 and EQU202503020056).

## CONFLICT OF INTEREST STATEMENT

There are no relevant conflicts of interest to declare.

## ETHICS STATEMENT

This study was approved by our ethics committee (CER‐BDX 2026‐23), and the database was analysed on 19 December 2025.

## Supporting information


**Table S1.** Definitions in the paediatric OBS'CEREVANCE cohort (actualization January 2026, date of start 2004, ongoing).

## Data Availability

Data presented in this manuscript are available from the corresponding author upon reasonable request.
