# Supplementary material for: Paediatric‐onset autoimmune cytopenia: How can we reduce the long‐term mortality?
Source: Br J Haematol. 2026 Jun 9;209(2):825–9. doi: 10.1111/bjh.70599 (PMC13461972; doi:10.1111/bjh.70599)
Supplement: Supplementary file 1 [file BJH-209-825-s001.docx]

**Table S1.** Definitions in the pediatric OBS’CEREVANCE cohort (actualization January 2026, date of start 2004, ongoing)

**Diagnosis criteria for auto-immune cytopenia (AIC): immune thrombocytopenia (ITP), autoimmune hemolytic anemia (AIHA), auto-immune neutropenia (AIN) and Evans syndrome (ES)**

**ITP**: according to the International Working Group criteria (Rodeghiero *et al*., *Blood* 2009).

**Chronic ITP:** ITP lasting > 12 months, according to the International Working Group criteria (Rodeghiero *et al*., *Blood* 2009).

**AIHA**: Hb < 110 g/L, with a positive direct antiglobulin test and at least one of the following hemolysis criteria: reticulocyte count > 120 G/L, free bilirubin > 17 mmol/L, or haptoglobin < 10 mg/dL.

**AIN:** peripheral neutropenia < 1.5 G/L intermittent or persisting for > 6 months, not explained by infection or drug use, regardless of the presence or absence of anti-neutrophil autoantibodies.

**Evans syndrome (ES):** simultaneous (< 1 month) or sequential association of 2 or 3 AIC, ITP, AIHA, and/or AIN.

**Inclusion criteria**

Patients are included in the OBS’CEREVANCE cohort if they were under 18 years old at the time of first diagnosis of AIC.

Follow-up and data collection are prospective from the initial diagnosis and continues into adulthood after the transfer in adult teams.

Follow-up duration is calculated from the date of diagnosis of the first AIC to the date of last follow-up.

**Exclusion criteria**

Confirmed inherited red cell or platelet disease.

**Clinical immunopathological manifestations (cIM)***

**Lymphoproliferation:** significant splenomegaly without hemolysis or significant persistent lymphadenopathy (> 1 cm), with or without biopsy.

**Autoimmune/autoinflammatory organ manifestations:** pulmonary (granulomatous-lymphocytic interstitial lung disease…), liver and digestive tract (lymphoid enteropathy, coeliac-like disease, inflammatory bowel disease, chronic gastritis, autoimmune or giant cell hepatitis, sclerosing cholangitis, cirrhosis…), central or peripheral nervous system (autoimmune, autoinflammatory, granulomatous, or vascular manifestations…), endocrine (autoimmune Hashimoto thyroiditis, Grave’s / Basedow disease…), dermatologic (psoriasis, vitiligo, alopecia, alopecia, vasculitis…), other (rheumatologic, renal, ophthalmologic, cardiac….).

**Severe allergy:** cutaneous (eczema, atopic dermatitis), ophthalmologic, ear-nose-throat (ENT), pulmonary, drugs or food

**Cancer, hematologic malignancies**

**Biological immunopathological manifestations (bIM)***

**Hypogammaglobulinemia**: immunoglobulin (Ig) G levels below the mean for the patient’s age (at least 2 SD) on two separate samples, associated with pyogenic infections, variably associated with IgA or IgM levels below the mean for the patient’s age (at least 2 SD), with defects in vaccine immunization, and/or with defects in B memory lymphocytes

**Autoimmune lymphoproliferative syndrome biomarkers:** elevated vitamin B12, persistent hypergammaglobulinemia (≥ 2 SD higher than the mean for age), high counts of circulating TCRαβ CD4− CD8− double-negative T lymphocytes, or increased circulating FAS ligand

**Systemic lupus erythematosus biomarkers:** antinuclear antibody titer ≥ 1/160; anti-double-stranded DNA antibodies, hypocomplementemia, lupus anticoagulant and / or anticardiolipin and / or lupus anticoagulant (antiphospholipid syndrome), elevated interferon signature

**Other isolated autoantibodies** (anti-thyroglobulin, anti-thyroperoxydase, anti-mitochondria, anti-transglutaminase…).

**Significant lymphopenia for age (global, CD4, CD8)** without any causative virus or immunosuppressive treatment

**Primary AIC**

Isolated ITP, AIHA, AIN or ES, absence of associated IM or causative disease during the follow-up (Rodeghiero *et al*., *Blood* 2009)

**Confirmed-secondary AIC****

Bone marrow or organ transplantation induced AIC, confirmed drug induced AIC

Primary ImmunoDeficiency (PID) / Inborn Error of Immunity (IEI) / Primary ImmunoDeficiency and Dysregulation (PIDD) genetically confirmed :

relevant phenotype and pathogenic validated variant in immune gene (class 4 or 5) (Poli et al*, JHI* 2025)

Systemic Lupus Erythematosus (SLE) (SLICC / EULAR criteria) (Petri et al, *Arthritis Rheum* 2012; Aringer et al, *Arthritis Rheum* 2019)

NB : infections (mycoplasma, EBV…) are frequent triggers in initial diagnosis or flares, they are not sufficient to classify AIC as secondary

**Likely-secondary AIC**

Any associated cIM or bIM before or after AIC occurrence throughout the available follow-up

Variants of unknown significance (VUS) or likely pathogenic (class 2, 3), in immune genes not yet demonstrated as conclusive for PID/IEI/PIDD

*French guidelines: <https://www.has-sante.fr/jcms/c_1340879/fr/protocoles-nationaux-de-diagnostic-et-de-soins-pnds>

1) in chronic AIC, according to age and sex at initial diagnosis, long term clinical and/or biological workup is recommended at least annually; the biological workup according to the clinical situation and guidelines, at the clinician’s discretion;

2) severe, repeated or opportunistic infections (viruses, bacteria, fungi, parasites, mycobacteria), and chronic organ infections (ENT, lung, digestive…), are attentively recorded, as they can be treatment related, or linked to an underlying immunodeficiency

3) genetic studies by Sanger, New Generation Sequencing, Whole Exome or Whole Genome sequencing are realized when the context is suggestive of PID/IEI/PIDD (familial or personal dysimmune anamnesis, resistance to treatments), depending on the clinician choice, multidisciplinary consultations and periods

**Second-line treatments**

First line treatments are corticosteroids and intravenous immunomodulatory immunoglobulins (dosage 0.8-1 g/kg).

Intravenous or subcutaneous replacement immunoglobulins (dosage 0.4-0.5 g/kg) are not considered as first-line nor second-line treatments.

Second-line treatments are splenectomy, immunomodulatory or immunosuppressive treatments (rituximab and other anti-B, hydroxychloroquine, azathioprine, cyclosporine, mycophenolate-mofetil, m-tor inhibitors, abatacept, JAK inhibitors, leniolisib…), thrombopoietin receptor agonists (eltrombopag, romiplostim)

Ancient or rarely used treatments are classified as “others” : anti-D, danazol, colchicine, vincristine, vinblastine…as anti-inflammatory biotherapies not directly indicated for AIC (anti-TNF, anti-IL1, IL6, …)

**ITP and AIHA status, at each visit, with the precision on-therapy or off-therapy**

No remission (NR) = platelet count < 30 G/L, Hb < 70 g/L;

Partial remission (PR) = platelet count 30–100 G/L, Hb 70–110 g/L or reticulocytes > 120 G/L;

Complete remission (CR) = platelet count > 100 G/L, Hb ≥ 11 g/L and reticulocytes ≤ 120 G/L

Continuous complete remission (CCR): no flare, no first-line, no second-line in the last 12 months.
